# VDR Gene Single Nucleotide Polymorphisms and Autoimmunity: A Narrative Review

**DOI:** 10.3390/biology12070916

**Published:** 2023-06-26

**Authors:** Cristina Agliardi, Franca Rosa Guerini, Elisabetta Bolognesi, Milena Zanzottera, Mario Clerici

**Affiliations:** 1 IRCCS Fondazione Don Carlo Gnocchi ONLUS, LAMMB, 20148 Milan, Italy; cagliardi@dongnocchi.it (C.A.); ebolognesi@dongnocchi.it (E.B.); mzanzottera@dongnocchi.it (M.Z.); mario.clerici@unimi.it (M.C.); 2 Department of Pathophysiology and Transplantation, University of Milan, 20122 Milan, Italy

**Keywords:** VDR, Vitamin D, SNP, autoimmunity, *ApaI*, *BsmI*, *TaqI*, *FokI*, multiple sclerosis, systemic lupus erythematosus

## Abstract

**Simple Summary:**

The pathogenesis of autoimmune diseases etiology is still mostly unclear and probably arises from an interplay between common/rare genetic variants and environmental factors that act as triggers. Vitamin D and its pathways have been repeatedly associated with the onset of autoimmune diseases. Vitamin D exerts its functions by binding to the Vitamin D receptor (VDR); the complex Vitamin D/VDR regulates many biological functions, including immune responses. In this review, we summarize and discuss data analyzing the possible involvement of the four best studied *VDR* gene polymorphisms in the pathogenesis of autoimmune conditions, with the aim of better understanding these mechanisms and shedding light on what needs to be further investigated.

**Abstract:**

The vitamin D/Vitamin D receptor (VDR) axis is crucial for human health as it regulates the expression of genes involved in different functions, including calcium homeostasis, energy metabolism, cell growth and differentiation, and immune responses. In particular, the vitamin D/VDR complex regulates genes of both innate and adaptive immunity. Autoimmune diseases are believed to arise from a genetic predisposition and the presence of triggers such as hormones and environmental factors. Among these, a role for Vitamin D and molecules correlated to its functions has been repeatedly suggested. Four single nucleotide polymorphisms (SNPs) of the *VDR* gene, *ApaI*, *BsmI*, *TaqI*, and *FokI*, in particular, have been associated with autoimmune disorders. The presence of particular *VDR* SNP alleles and genotypes, thus, was observed to modulate the likelihood of developing diverse autoimmune conditions, either increasing or reducing it. In this work, we will review the scientific literature suggesting a role for these different factors in the pathogenesis of autoimmune conditions and summarize evidence indicating a possible *VDR* SNP involvement in the onset of these diseases. A better understanding of the role of the molecular mechanisms linking Vitamin D/VDR and autoimmunity might be extremely useful in designing novel therapeutic avenues for these disorders.

## 1. Introduction

### 1.1. Vitamin D and VDR

Vitamin D is a micronutrient with a fundamental role in human health. It is produced in the skin from 7-dehydrocholesterol after exposure to ultraviolet B radiation (wavelength: 290–315 nm) and, only partially (20%), can be obtained from diet (cod-liver oil, salmon, tuna, trout, liver, eggs, etc.). The biologically active form of Vitamin D is 1,25-dihydroxyvitamin D_3_ [1,25-(OH)_2_D_3_] (calcitriol), which is produced through progressive hydroxylation in the liver and kidneys; however, hydroxylation can be performed in other tissues and cells, including epithelial cells, parathyroid glands, and macrophages. The hydroxylation process is modulated by tumor necrosis factor α (TNFα) and interferon γ (IFNγ) cytokines [1]. 1,25-(OH)_2_D_3_ is a secosteroid hormone that exerts its function through binding to the vitamin D receptor (VDR) [2,3]. VDR was identified in 1974 [4,5] and is responsible for the activation of the genomic pathway of 1,25-(OH)2D3. Briefly, upon binding Vitamin D, VDR becomes a ligand for an activated, DNA-binding transcription factor, which generates an active signal transduction complex. Such a complex includes a Vitamin D/VDR heterodimer and the retinoid X receptor (RXR), and it translocates into the cell nucleus where it recognizes the vitamin D responsive elements (VDREs) positioned in the DNA sequence of Vitamin D-regulated genes [6,7]. It has nevertheless to be underlined that a potential non-genomic activity of the VDR that could cause transcription-independent responses in cells has been hypothesized as well [8]. The nearly ubiquitous expression of VDR suggests that the Vitamin D/VDR axis regulates genes involved in different functions: calcium homeostasis [9], energy metabolism, cell growth and differentiation, and immune responses [10,11,12]. In particular, VDR regulates genes of innate and adaptive immunity [13].

### 1.2. Vitamin D/VDR and Autoimmunity

**Figure 1:**
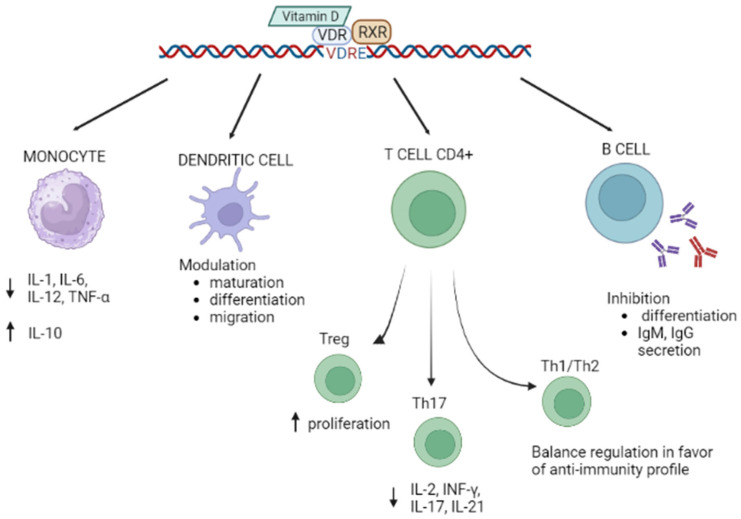
Regulation of immune processes by the axis Vitamin D/VDR. Up arrows: increase, down arrow: decrease. Created with BioRender.com.

Low levels of Vitamin D, together with genetic variations within the *VDR* gene, have been associated with susceptibility to different conditions, including neurodegenerative diseases [14], neurodevelopmental diseases [15], frailty in elders [16], and, particularly, autoimmune diseases [7,17]. This review will focus on the possible role played by Vitamin D in the pathogenesis of autoimmune conditions. Autoimmune disorders are believed to arise from a genetic predisposition interacting with triggers including hormones, infections, and environmental factors [18]. A role for Vitamin D in the regulation of innate and adaptive immune responses was suggested by the discovery that VDR is expressed by almost all immune cells (activated CD4+ and CD8+ T cells, B cells, neutrophils, antigen-presenting cells (APC), dendritic cells, and macrophages). Notably, Vitamin D was described to inhibit the production of pro-inflammatory cytokines IL-1, IL-6, IL-12, and TNFα and increase that of the anti-inflammatory cytokine IL-10 by monocytes and to modulate the differentiation and maturation of dendritic cells [19,20]. Vitamin D, thus, may interact directly with dendritic cells, influencing both their migration and their ability to initiate and functionally differentiate T lymphocytes, triggering and modulating immune responses [21]. Vitamin D can also promote the proliferation and function of immunosuppressive Treg cells [22] and plays a role in Th1/Th2 balance, limiting inflammation and autoimmunity [23]. Importantly, this vitamin also modulates the functions of Th17 cells, a T cell subset of pivotal importance in autoimmunity [24], down-regulating the release of the proinflammatory cytokines IL-2, INF-γ, IL-21, and IL-17 [25]. Finally, Vitamin D inhibits the differentiation of B lymphocytes into plasma cells and antibody production and greatly reduces the formation of B memory cells and immunoglobulin secretion by activated B cells [26] (Figure 1).

### 1.3. VDR Gene Structure and Principal Polymorphisms

The *VDR* gene is located on chromosome 12q13.11 and comprises 14 exons. In the promoter region lies exon 1, which has six variants (from *a* to *f*), important for alternative splicing; in the coding region are present exons 2–9, common for all 14 transcripts [27]. Only three different isoforms have been described in human cells:

- VDRA: start site in exon 2, 427 amino acids, 48 kDa;
- VDRB1: start site in exon 1d, 477 amino acids, 54 kDa;
- a shorter isoform with higher transcriptional activity is created by a *FokI* single nucleotide polymorphism (SNP) that creates a translation initiation codon [28]: 424 amino acids, 47 kDa.

**Figure 2:**
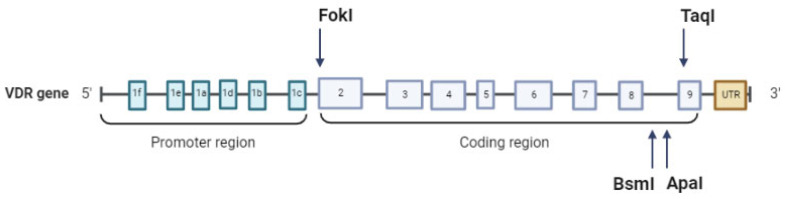
*VDR* gene structure. The most common SNPs, *FokI* (rs2228570), *BsmI* (rs1544410), *ApaI* (rs7975232), and *TaqI* (rs731236), are shown. Created with BioRender.com.

The *VDR* gene harbors more than 900 allelic variants [29], a fraction of which is believed to interfere with Vitamin D function. We will focus on the most investigated of these allelic variants. In intron 8/exon 9, at the 3’ end of the *VDR* gene, there are three adjacent SNPs that have been extensively studied in correlation with different pathologies: rs1544410 +63980 (*BsmI*, C > T(B > b)), rs7975232 + 64978 (*ApaI*, A > C (A > a)), and rs731236 (*TaqI* + 65058 (T > C (T > t)). Initially detected by the restriction fragment length polymorphism (RFLP) technique, the VDR genotype was identified by the presence (b, a, and t) or absence (B, A, and T) of the *BsmI*, *ApaI*, and *TaqI* cleavage sites [15]. Another important SNP already mentioned above is rs2228570 (*FokI* + 30920 C > T(F > f)), located in exon 2. The T-to-C substitution eliminates the first ATG translation initiation site, determining a final protein of 424 amino acids instead of the 427 amino acids of the wild-type product. This shorter form is characterized by augmented transcriptional activation [28] (Figure 2).

*BsmI* and *ApaI* SNPs are associated with decreased stability of mRNA and lower expression levels; *TaqI*, near the exon-intron boundary, might affect splicing and thus VDR translation [30] (Table 1) (Figure 2).

The genetic models used to study *ApaI*, *BsmI*, *FokI*, and *TaqI* are summarized in Table 2.

## 2. VDR Polymorphisms and Principal Autoimmune Diseases

### 2.1. Multiple Sclerosis

Multiple sclerosis (MS) is a chronic autoimmune demyelinating disease of the central nervous system (CNS) characterized by the loss of sensory and motor function and major clinical disabilities [31]. MS affects 2 million people worldwide. As with other immune-mediated disorders, MS has a complex pathophysiology that involves genetic susceptibility and unknown environmental triggers [32]. The major genetic risk factor for MS is the HLA-DRB1*15 allele [33,34], but over 200 minor genetic risk variants, all SNPs, have now been described [35].

VDR SNPs have been widely investigated in MS. A meta-analysis [36] analyzed a total of 4013 MS cases and 4218 controls in 24 case-control studies. The results indicate no association between *BsmI*, *ApaI*, *TaqI*, and *FokI* among overall populations of Asians and Caucasians; the A allele of *ApaI* alone resulted in being associated with MS risk in Asian populations (596 MS cases/731 HC) (*p* = 0.005, OR = 1.27, 95% CI = 1.07–1.50). Another meta-analysis [37], which included 3758 cases and 3992 HC of European and Asian origin, indicated an involvement of *TaqI* under the heterozygote model (CT vs. TT) (*p* < 0.0001, OR = 1.27, 95% CI = 1.01–1.59). A third meta-analysis [38] analyzed nine case-control studies for an overall population of 1206 MS Iranian cases and 1402 Iranian HC. *TaqI* was studied in 721 MS cases and 696 HC cases. A significant negative association with MS susceptibility emerged under the homozygote (TT vs. CC) genetic model (*p* = 0.04, OR = 0.28, 95% CI = 0.08–0.94). *ApaI* was evaluated in 721 MS cases and 696 HC cases. The allelic genetic model revealed a significant association between the A allele and a decreased risk of MS, A vs. C (*p* < 0.01, OR = 0.54, 95% CI = 0.37–0.79), the same under the homozygote genetic model (AA vs. CC) (*p* < 0.01, OR = 0.28, 95% CI = 0.14–0.59). *FokI* was evaluated in 576 MS cases and 738 HC, but no association with MS risk emerged under all the genetic models (an explanation of the genetic models used is reported in Table 2). Similarly, *BsmI*, evaluated in 738 MS patients and 738 HC, revealed no association with MS susceptibility. Finally, a study performed in 641 Italian MS patients and 558 HC showed a protective role against MS risk for the VDR *TaqI* T allele-HLA-DRB1*15+ haplotype (*p* = 9.5 × 10^−5^; OR = 2.52; 95% CI = 1.56–4.06) and, on the contrary, an increased risk of developing MS associated with the -DRB1*15-*VDR TaqI* C haplotype. *TaqI* TT genotype was also protective in HLA-DRB1*15-positive subjects (*p* = 0.004; OR = 0.53; 95% CI = 0.33–0.83) [39] (Table 3). In summary, multiple pieces of evidence support a role for the *TaqI* VDR SNP in MS susceptibility.

### 2.2. Behcet’s Disease

Behcet’s disease (BD) is a chronic autoimmune multisystemic disorder. It is an inflammatory vasculopathy with an unknown etiology. A strong genetic correlation with the human leukocyte antigen (HLA)*B51 allele has been described [40]. A higher prevalence of the disease is present in the Middle East, the Far East, and the Mediterranean [41]. Much attention has been paid to the possible correlation between BD and *VDR* SNPs. A recent meta-analysis took into consideration seven independent studies for a total of 478 cases and 666 HC [42]. Pooled results indicated a significant association between *ApaI* and BD risk in all populations: allelic model (A vs. C) (*p* = 0.001, OR = 1.382, 95% CI = 1.142–1.672). A more noticeable effect was found in the Caucasian subgroup: homozygote model (AA vs. CC) (*p* = 0.008, OR = 2.616, 95% CI = 1.284–5.329), suggesting a correlation between the *ApaI* AA genotype and BD development. *BsmI*, *TaqI*, and *FokI* polymorphisms were not correlated with BD in overall analyses, but strong relationships were found in ethnicity-based subgroups. For *BsmI*, an association with BD risk was observed in Caucasians (200 BD cases, 200 HC) under the allelic model: C vs. T (*p* = 0.04, OR = 1.34, 95% CI = 1.014–1.770) and under the recessive model: CC vs. CT + TT (*p* = 0.047, OR = 1.561, 95% CI = 1.006–2.421). For *TaqI* (Table 3), a protective role was found among Africans (188 cases, 239 HC) under the allelic model T vs. C (*p* = 0.034, OR = 0.742, 95% CI = 0.563–0.978). Similarly, *FokI* was associated with BD in Africans (176 cases, 197 HC) under the allelic model T vs. C (*p* < 0.001, OR = 0.548, 95% CI = 0.405–0.741) and the dominant model TT + CT vs. CC (*p* = 0.001, OR = 0.326, 95% CI = 0.171–0.622) (Table 3).

The possible *VDR* SNP involvement in Behcet’s disease susceptibility needs to be investigated in larger cohorts of various ethnicities, as the results of the meta-analysis we summarized were based on a relatively small sample size of cases and controls.

### 2.3. Systemic Lupus Erythematosus (SLE)

Systemic lupus erythematosus (SLE) is an autoimmune, multifactorial disease caused by a combination of genetic susceptibility and environmental factors [43]. Genome-wide association studies (GWAS) revealed more than 40 genetic loci associated with susceptibility to SLE, among them *VDR* [44]. A recent meta-analysis by Yang et al. [45] evaluated the results of 19 papers: 6 concerning *ApaI* (rs7975232), 15 concerning *BsmI* (rs1544410), 12 concerning *FokI* (rs2228570), and 4 concerning *TaqI* (rs731236). Overall, 2663 patients and 3252 control subjects of different ethnicities were studied. Results showed that *ApaI* (rs7975232) was correlated with SLE susceptibility in the overall populations (AA vs. CC: OR = 1.374, 95% CI: 1.115–1.692, *p* = 0.003; AA + AC vs. CC: OR = 1.342, 95% CI: 1.139–1.583, *p* < 0.01), and in particular in Caucasian (AA vs. CC: OR = 1.329, 95% CI: 1.016–1.740, *p* = 0.038) and Asian (AA + AC vs. CC: OR = 1.351, 95% CI: 1.043–1.749, *p* = 0.023) patients. Moreover, a higher-risk link between *ApaI* (rs7975232) polymorphism and SLE susceptibility was detected to be present in females (AA vs. CC: OR = 1.392, 95% CI = 1.049–1.849, *p* = 0.022). In contrast, no correlation between *BsmI* (rs1544410) and SLE susceptibility was observed in the overall populations, but stratification by race allowed to detect significant associations both for Caucasians (913 SLE cases/1271 HC) (CC vs. CT + TT: OR = 0.734, 95% CI: 0.593–0.909, *p* = 0.005; C vs. T: OR = 0.865, 95% CI: 0.760–0.983, *p* = 0.026) and Africans (241 SLE cases/245 HC) (CC + CT vs. TT: OR = 2.935, 95% CI: 1.944–4.430, *p* < 0.01; C vs. T: OR = 1.898, 95% CI: 1.458–2.470, *p* < 0.01). *FokI* (rs2228570) was associated with SLE susceptibility in African populations alone (441 cases/445 HC) (CC vs. CT + TT: OR = 2.424, 95% CI: 1.673–3.512, *p* < 0.01; C vs. T: OR = 1.720, 95% CI: 1.417–2.087, *p* < 0.01; CC vs. TT: OR = 3.154, 95% CI: 2.083–4.774, *p* < 0.01; CC + CT vs. TT: OR = 1.803, 95% CI: 1.363–2.384, *p* < 0.01). Finally, no association between *TaqI* (rs731236) and SLE was reported (Table 3).

The involvement of *VDR* SNPs, especially *ApaI*, in SLE susceptibility seems to be demonstrated by these results, but the exact mechanism by which these *VDR* SNPs play a role in the pathogenesis of SLE needs to be further investigated. The observed sex-associated differences will need to be better clarified as well.

### 2.4. Type 1 Diabetes

Type 1 diabetes (T1D) is a chronic T-cell-mediated autoimmune disease characterized by the destruction of β-cells in pancreatic islets of Langerhans that results in insulin deficiency and hyperglycemia [46]. T1D affects more than half a million individuals in the world, and almost 90,000 children have a T1D diagnosis each year [47]. T1D is a polygenic disease, with multiple predisposing and protective alleles interacting with each other [48]. Numerous association studies of VDR polymorphisms and T1D have been conducted. A meta-analysis [49] included 23 papers overall. Analyzed individually, none of the *BsmI*, *ApaI*, *TaqI*, or *FokI* SNPs resulted in associations with T1D risk. A protective role against T1D was found instead for the *BsmI-ApaI-TaqI* b-A-T (T-A-T) haplotype (*p* = 0.007, OR = 0.639, 95% CI = 0.460–0.887).

A more recent meta-analysis [50] included 39 papers. *FokI* was analyzed in 3723 T1D cases and 5578 HC. The pooled results showed no significant association with the overall population. However, ethnicity-based subgroup analyses revealed an association with T1D in Europeans (1960 T1D cases/3715 HC) under the dominant model (TT + CT vs. CC) (*p* = 0.05, OR = 0.86, 95% CI = 0.74–1.00) and heterozygote contrast (CT vs. CC) (*p* = 0.04, OR = 0.86, 95% CI = 0.75–0.99). In Africans (312 cases/220 HC), an increased T1D susceptibility was found under all genetic models: dominant (TT + CT vs. CC) (*p* = 0.008, OR = 2.06, 95% CI = 1.20–3.53), recessive (TT vs. CT + CC) (*p* = 0.04, OR = 2.14, 95% CI = 1.03–4.43), allelic model (T vs. C) (*p* = 0.02, OR = 1.17, 95% CI = 1.06–2.97), homozygote (TT vs. CC) (*p* = 0.004, OR = 3.11, 95%CI = 1.44–6.69), and heterozygote (CT vs. CC) (*p* = 0.01, OR = 1.81, 95% CI = 1.13–2.91). *TaqI* was investigated in 1837 T1D cases and 1895 HC and was not associated with T1D risk. *BsmI* was investigated in 4826 T1D patients and 7159 HC patients and was not found to be associated with either T1D or HC under all genetic models for the overall population. Analyses of ethnicity-based subgroups, though, showed a role for *BsmI* in T1D in American populations (463 T1D cases/479 HC): dominant model (TT + CT vs. CC) (*p* = 0.004, OR = 0.57, 95% CI = 0.39–0.84), recessive model (TT vs. CT + CC) (*p* = 0.02, OR = 0.62, 95% CI = 0.41–0.94), allelic model (T vs. C) (*p* < 0.001, OR = 0.66, 85% CI = 0.54–0.81), homozygote model (TT vs. CC) (*p* = 0.003, OR = 0.52, 95% CI = 0.34–0.80). Finally, *ApaI* was analyzed in 2436 T1D cases and 4074 HC, and no associations were found with T1D (Table 3).

To summarize, even if no significant association of *VDR* gene SNPs with T1DM risk was detected in the overall population, subgroup analysis showed the presence of significant associations between *FokI* and *BsmI* polymorphisms and T1DM risk in the African and American populations.

### 2.5. Celiac Disease

Celiac disease (CD) is an autoimmune disease that occurs in genetically predisposed individuals. The main genetic risk factor is the presence of the *HLA-DQ2-HLA-DQ8* alleles. CD is characterized by a massive pro-inflammatory immune response against certain parts of gluten and the intestinal tissue itself, resulting in structural changes. The clinical manifestations are broad and include extra-intestinal symptoms [51]. CD patients have lower Vitamin D levels but higher levels of 1,25-(OH)_2_D_3_ than HC patients [52]. A recent review [53] collected data from four papers that investigated VDR SNPs in CD (176 CD and 402 HC). *FokI* T allele was associated with a higher CD risk (*p* = 0.02, OR = 1.52, 95% CI = 1.06–2.18), whereas *ApaI*, *BsmI*, and *TaqI* SN*Ps* were not associated with CD (Table 3).

### 2.6. Vitiligo

Vitiligo is an autoimmune chronic skin disease affecting 0.5–2% of the population [54] and characterized by depigmentation due to selective melanocyte loss in the affected areas of the skin.

The lesions evolve over time and are typically distributed in an acro-facial pattern (periorificial facial and hands/feet involvement) or scattered symmetrically over the entire body. The etiopathogenesis of vitiligo is still unknown, but it is believed that the initial event is an intrinsic defect of melanocytes that leads to oxidative stress, local inflammation, and activation of the innate immune response, which generate melanocyte-specific cytotoxicity in the presence of genetic predisposition.

Zhang JZ [55] evaluated possible associations between *ApaI*, *BsmI*, *TaqI*, and *FokI VDR* SNPs, serum 25 (OH)D, and the risk of vitiligo by analyzing 17 articles. For the *ApaI* SNP, the meta-analysis was applied to a population of 1250 cases and 1400 HC. A significant statistical association was observed under the dominant genetic model (CC + AC vs. AA, *p* = 0.007, OR = 1.41, 95% CI = 1.10–1.80), the recessive genetic model (CC vs. AC + AA, *p* = 0.01, OR = 4.10, 95% CI = 1.36–12.35), and the allelic model (C vs. A, *p* = 0.005, OR = 1.87, 95% CI = 1.21–2.90). No association was found between the other VDR SNPs, *BsmI*, *TaqI*, and *FokI*, and vitiligo.

A more recent meta-analysis [56] analyzed 13 papers. A protective role for the *ApaI* A allele (1048 cases and 1058 HC) (*p* = 0.016, OR = 0.721, 95% CI = 0.553–0.940) and the *BsmI* C allele (849 cases and 843 HC) (*p* = 0.015, OR = 0.812, 95% CI = 0.686–0.961) was detected in Asiatic populations alone (Table 3). These results highlight once again the importance of stratification by ethnicity when doing such studies.

### 2.7. Psoriasis

Psoriasis is an autoimmune, chronic, inflammatory, and multisystemic disease that affects predominantly the skin and joints. It is caused by the same complex interplay between genetic and environmental factors as other autoimmune conditions [57]. A meta-analysis [58] analyzed the results of 18 case-control studies. *ApaI*, *BsmI*, FokI, and *TaqI* SNPs were not associated with psoriasis susceptibility, but the authors stated that more case-control studies are needed to shed light on VDR SNPs involvement with psoriasis. A previous meta-analysis [59] indicated a possible role for *TaqI* in Caucasian populations (205 cases and 550 HC) (TT genotype, *p* = 0.046, OR = 1.29, 95% CI = 1.00–1.66) but not in Asians. (Table 3). To summarize, more data are needed as the possible positive association of *TaqI* with psoriasis in Caucasians is based on a relatively small cohort of subjects.

### 2.8. Rheumatoid Arthritis (RA)

RA is a common autoimmune disease characterized by the production of autoantibodies, chronic synovial inflammation, and progressive destruction and deformity of joints [60]. Genetic factors contribute to about 50–65% of the RA risk [61]. Several association studies between *VDR* gene polymorphisms and RA risk yield conflicting results. A recent meta-analysis [62] included the results of 23 studies.

*FokI* SNP was analyzed in 2170 cases and 2452 HC. The pooled OR detected a protective association between *FokI* SNP and susceptibility to RA under the dominant model (TT + CT vs. CC, *p* < 0.001, OR = 0.74, 95% CI = 0.60–0.92), the TT vs. CC model (*p* < 0.001, OR = 0.66, 95% CI = 0.54–0.81), and the CT vs. CC model (*p* < 0.001, OR = 0.85, 95% CI = 0.73–0.98), but not the allelic (C vs. T) and recessive model (TT vs. CT + CC). There were some differences between different ethnic groups, as no associations were found between Africans and Arabs.

*TaqI* SNP was analyzed overall in 1334 cases and in 1560 HC in studies conducted in Europeans, Asians, and Africans. Pooled results showed no associations between *TaqI* SNP and RA risk. However, subgroup analyses revealed a protective role of *TaqI* in Africans (339 RA cases/383 HC): dominant model (CC + CT vs. TT) (*p* = 0.01, OR = 0.50, 95% CI = 0.29–0.85), recessive model (CC vs. CT + TT) (*p* < 0.001, OR = 0.44, 95% CI = 0.25–0.79), allelic (C vs. T) (*p* = 0.001, OR = 0.57, 95% CI = 0.37–0.88), CC vs. TT (*p* < 0.001, OR = 0.32, 95% CI = 0.15–0.72), CT vs. TT (*p* < 0.001, OR = 0.57, 95% CI = 0.38–0.87). Similar results were obtained in Arab populations (191 RA cases/246 HC): recessive model (CC vs. CT + TT) (*p* = 0.01, OR = 0.53, 95% CI = 0.32–0.87) and CC versus TT (*p* = 0.03, OR = 0.43, 95% CI = 0.20–0.94). No associations were found either in European or Asian cohorts.

*BsmI* SNP was studied in an overall population of 2153 cases and 2326 HC that included Europeans, Asians, and Africans. Pooled results revealed no significant association between *BsmI* and the risk of RA. Analyses conducted in ethnic subgroups showed a positive association in Africans (541 RA cases/532 HC) under all genetic models: dominant model (TT + CT vs. CC) (*p* = 0.01, OR = 1.82, 95% CI: 1.14–2.88), recessive model (TT vs. CT + CC) (*p* = 0.01, OR = 1.77, 95% CI = 1.13–2.78), allelic (T vs. C) (*p* < 0.001, OR = 1.59, 95% CI = 1.14–2.23), TT vs. CC (*p* = 0.01, OR = 2.40, 95% CI = 1.22–4.71), and CT vs. CC (*p* = 0.02, OR = 1.45, 95% CI = 1.04–2.01). No associations were found between the *BsmI* SNP and RA risk in Europeans, Asians, or Arabs.

*ApaI* SNP was studied in a total of 1191 cases and 1415 HC, and the polymorphism was significantly associated with RA risk only under the AC vs. AA model (*p* = 0.01, OR = 0.76, 95% CI = 0.61–0.94).

*FokI* and *Taq I* VDR SNPs, thus, were significantly associated with the risk of developing RA both in overall and subgroup analyses. A post-2020 study [63] confirmed FokI SNP involvement in RA risk in the Serbian population. Finally, a study on Italians [64] (191 RA patients and 246 HC) confirmed a protective role for the *TaqI* CC genotype against RA risk (*p* = 0.045, OR = 0.55, 95% CI = 0.31–0.99) (Table 3). *TaqI* involvement in the pathogenesis of RA, thus, seems to be confirmed by several studies.

### 2.9. Systemic Sclerosis

Systemic sclerosis (SS) is a systemic autoimmune rheumatic disease characterized by endothelial dysfunction, leading to small vessel vasculopathy, fibroblast dysfunction, and subsequent fibrosis of the skin and viscera and immune dysregulation [65]. *VDR* SNPs were investigated only in two studies that gave conflicting results. Kamal et al. found that *ApaI* and *TaqI* SNPs were not associated with SS in a very small cohort of Egyptian subjects (30 SS patients and 60 healthy controls) [66]. Li et al. studied 8 VDR SNPs (*TaqI*, *FokI*, *ApaI*, BsmI, rs11574010 (*Cdx2*), rs739837 (*BglI*), rs757343 (*Tru9I*), and rs11168267) in 100 SS patients and 100 Han Chinese HC; results showed that SS risk was higher in *ApaI* SNP AA + AC genotype carriers than in CC genotype carriers (dominant model). Similarly, CT + TT genotypes of the VDR *BglI* (rs739837) polymorphism were associated with increased risk of SS when compared to the CC genotype (dominant model) [67] (Table 3). These results are based on extremely small cohorts and need to be confirmed in ampler studies.

### 2.10. Sjögren Syndrome

Primary Sjögren syndrome (SjS) is a chronic and systemic autoimmune disease characterized by lymphocytic infiltrates of the exocrine organs, including the lacrimal, salivary, and parotid glands, leading to dryness symptoms, parotid enlargement, fatigue, and pain [68]. Notably, 30–40% of SjS patients will develop systemic manifestations such as skin, joints, muscles, kidneys, liver, lungs, heart, and peripheral and central nervous system abnormalities [69]. As is the case with all other autoimmune diseases, the etiology of SjS is complex and is believed to stem from the interaction between genetic factors and various environmental triggers, such as viral infections [70]. A few studies have been conducted in order to verify the possible involvement of *VDR* polymorphisms in SjS. A study that involved 105 patients and 93 HC did not find any association between *BsmI*, *TaqI*, *ApaI*, and *FokI VDR* SNPs and SjS susceptibility [71]. These results were confirmed by a more recent study conducted on 195 SjS patients and 246 HC patients from Italy [64]. It nevertheless has to be underlined that very few subjects were tested; analyses in ampler cohorts are needed.

### 2.11. Hashimoto’s Thyroiditis (HT) and Grave’s Disease (GD)

Hashimoto’s thyroiditis (HT) is a chronic autoimmune lymphocytic thyroiditis due to the loss of self-tolerance against thyroid antigens that cause hypothyroidism [72]. HT is one of the most frequent organ-specific autoimmune diseases, affecting up to 5% of the population [73]. As for other autoimmune disorders, accumulating evidence indicates that HT is triggered by a synergistic contribution of genetic and environmental factors. A meta-analysis [74] evaluated the association between *VDR* SNPs and HT development in 11 articles, including 1338 overall HT cases and 1303 HC.

*FokI* was evaluated in 978 HT patients and 938 HC patients. Pooled results showed a significant association under the allelic model C vs. T (*p* = 0.010, OR = 1.44, 95% CI = 1.09–1.91) and dominant model (CC vs. TT + CT) (*p* = 0.019, OR = 1.72, 95% CI = 1.09–2.71) but not under other genetic models. The pooled results did not observe any correlations between *BsmI*, *ApaI*, and *TaqI* SNPs and HT susceptibility.

One study was published after the publication of this meta-analysis [75] and confirmed those results. One hundred twenty-one patients (suffering from either HT or Grave’s disease) and 117 H were analyzed. An association between CC and CT *FokI* genotypes and susceptibility to autoimmune thyroid diseases (*p* = 0.03, OR = 3.75; 95% CI, 1.16–12.16, and *p* = 0.04; OR = 3.41; 95% CI, 1.03–11.28, respectively) was noticed. In particular, the CC genotype was associated with HT risk (*p* = 0.04, OR = 3.38, 95% CI = 1.04–11). In this case, the confirmation of FokI involvement in HT risk is quite interesting.

Grave’s disease is another common T-cell-mediated autoimmune hyperthyroidism characterized by the presence of autoantibodies directed toward the thyroid-stimulating hormone receptor (TSHR), resulting in an excessive production of thyroid hormones [76]. Results of a meta-analysis that included 1820 GD patients and 2066 HC of Caucasian and Asian origin showed that *ApaI*, *BsmI*, and *FokI* VDR SNPs were associated with GD in Asians (300 GD/295 HC, 300 GD/297 HC, 219 GD/240 HC) under an allelic model (A vs. C: *p* = 0.02, OR = 1.31; 95% CI, 1.04–1.67; C vs. T: *p* = 0.007, OR = 1.58; 95% CI, 1.13–2.22; and C vs. T: *p* = 0.009, OR = 1.68; 95% CI: 1.14–2.59), respectively [77]. None of the VDR SNPs were associated with GD risk in Caucasians (Table 3). It is interesting that the *FokI* C allele was associated with both HT and GD; this suggests the presence of a common molecular basis for these two thyroid autoimmune diseases.

**Table 3:** The table summarizes the principal results for VDR SNPs *TaqI*, *BsmI*, *ApaI*, and *FokI* SNPs involvement in autoimmune diseases.

Disease	*ApaI* (Cases/Controls) Etnicity		*BsmI* (Cases/Controls) Etnicity		*FokI* (Cases/Controls), Etnicity		*TaqI* (Cases/Controls), Etnicity		Ref.
	risk	protection	risk	protection	risk	protection	risk	protection	
Multiple sclerosis	A vs. C (596/731) Asians								[36]
		A, AA vs. CC (721/696) Iranian						TT (721/696) Iranian	[38]
								T, TT (in HLADRB1*15+) (641/558) Italians	[39]
							CT vs. TT (3758/3992) overall		[37]
Behcet’s disease	A vs. C (478/666) overall AA vs. CC (237/230) Caucasians		C vs. T CC vs. CT + TT (200/200) Caucasians			T vs. C TT + CT vs. CC (176/197) Africans		T vs. C (188/239) Africans	[42]
Systemic lupus erythematosus	AA vs. CC (1320/1736) overall AA vs. CC females AA vs. CC (759/1019) Caucasians		C vs. T, CC + CT vs. TT (241/245) Africans	CC vs. CT + TT, C vs. T (913/1271) Caucasians	CC vs. CT + TT, C vs. T, CC vs. TT, CC + CT vs. TT (441/445) Africans				[45]
Type 1 diabetes				TT + CT vs. CC TT vs. CT+CC T vs. C TT vs. CC (463/479) Americans	TT + CT vs. CC TT vs. CT + CC T vs. C TT vs. CC CT vs. CC (312/220) Africans	TT + CT vs. CC CT vs. CC (2077/3849) Europeans			[50]
Celiac disease					T (176/402)				[53]
Vitiligo	CC + AC vs. AA CC vs. AC + AA C vs. A (1250/1400)								[55]
		A vs. C (1048/1058) Asians		C vs. T (849/863) Asians					[56]
Psoriasis							TT (205/550) Caucasians		[58]
Rheumatoid arthritis		AC vs. AA (1191/1451)	TT vs. CT + CC T vs. C TT vs. CC CT vs. CC (541/532) Africans			TT (2170/2452), overall		CC + CT vs. TT CC vs. CT + TT C vs. T CC vs. TT CT vs. TT (339/383) Africans CC vs. CT + TT CC vs. TT (560/623) Arabs	[62]
								CC (191/246) Italians	[64]
Hashimoto’s thyroiditis					C vs. T CC vs. TT + CT (978/938) overall				[74]
					CC (121/117)				[75]
Grave’s disease	A vs. C (300/295) Asians		C vs. T (300/297) Asians		C vs. T (219/240) Asians				[77]

## 3. Discussion

Autoimmune diseases are believed to be associated with a yet only partially understood genetic basis, and multiple genes are probably involved in the predisposition to each disease in a modest way [78]. Moreover, autoimmune conditions are believed to arise from an interplay between genetic and environmental factors.

The aim of this narrative review was to explore and discuss the possible role played by VDR and its four more widely investigated SNPs (*ApaI*, *BsmI*, *TaqI*, and *FokI*) in the pathogenesis of autoimmune diseases. The genetics of *VDR* are of particular significance for multifactorial diseases since the vitamin D/VDR axis itself is highly dependent on environmental factors such as diet and sun exposure. The four SNPs of *VDR* influence the function of the Vitamin D/VDR complex in multiple ways: *ApaI* and *FokI* modulate serum Vitamin D concentrations [79], *TaqI* impacts *VDR* expression [39], and *BsmI* influences INF-γ production by PBMC [80]. The biological effects of these SNPs, though, are still mostly unknown and need to be explored further. VDR SNPs were found to be associated with the pathogenesis of most of the autoimmune diseases reviewed here, and some risk and/or protective SNP alleles and genotypes appear to be shared by different autoimmune disorders. Thus, the *ApaI* A allele and AA genotype are risk factors shared by Multiple Sclerosis, Behcet’s disease, and Systemic lupus erythematosus. On the contrary, the *FokI* T allele and TT genotype seem to be protective factors for Bechet’s disease, Type I diabetes mellitus, and rheumatoid arthritis. The results of many of these case-control studies are often discordant [36,38]. This may be due to small sample sizes, low statistical power, the probable presence of false positive results, extensive ethnic variations, and interactions with other genetic or environmental factors. Probably because of this last reason, contrasting results have often been found in different ethnic-based subgroups. It also has to be underlined that reported associations in ethnic subgroups by some meta-analyses are based on very small sample sizes after stratification; this could introduce bias due to low statistical power [42]. Finally, we are aware that this review has some limitations, including biases in the literature search and selection.

## 4. Conclusions

We suggest designing future case-control studies taking into consideration only homogeneous populations from racial and ethnic points of view to better clarify the contribution of VDR SNPs to autoimmune diseases in different populations. Genotype distributions, in fact, may vary by ancestry at many loci. We would also recommend that future studies analyze the synergistic contribution of the VDR SNPs to autoimmune disease susceptibility by studying not only single SNPs alone but also haplotypes as well (see, e.g., [49]). Moreover, much effort will need to be spent in order to identify the mechanistic/functional effect of every SNP that contributes to the risk and prognosis of diseases. We also urge the publication of negative results to have a more complete picture of the real involvement of such genetic polymorphisms in the risk of autoimmune diseases and to decrease the incidence of false positive results.

## Figures and Tables

**Table 1:** VDR gene *TaqI*, *BsmI*, *ApaI,* and *FokI* SNP positions, descriptions, and effects.

*VDR* SNP	Position	Description	Effect
*TaqI* (rs731236)	+65,058, intronic	T > C (T < t)	Affects splicing and VDR translation
*BsmI* (rs1544410)	+63,980, intronic	C < T (B > b)	Decreases mRNA stability
*ApaI* (rs7975232)	+64,978, intronic	A > C (A > a)	Decreases mRNA stability
*FokI* (rs2228570)	+30,920, exon 2	C > T (F < f)	Start loss

**Table 2:** Description of the genetic models used to study *TaqI*, *BsmI*, *ApaI*, and *FokI* SNPs.

Genetic Model	*ApaI*	*BsmI*	*FokI*	*TaqI*
Dominant	CC + AC vs. AA	TT + CT vs. CC	TT + CT vs. CC	CC + CT vs. TT
Recessive	CC vs. AC + AA	TT vs. CT + CC	TT vs. CT + CC	CC vs. CT + TT
Heterozygote	AC vs. AA	CT vs. CC	CT vs. CC	CT vs. TT
Homozygote	CC vs. AA/AA vs. CC	TT vs. CC/CC vs. TT	TT vs. CC/CC vs. TT	CC vs. TT/TT vs. CC
Allelic	C vs. A/A vs. C	T vs. C/C vs. T	T vs. C/C vs. T	C vs. T/T vs. C

## Data Availability

No new data were created or analyzed in this study.
